# Cerebral Sinus Venous Thrombosis in the Setting of Acute Mastoiditis

**DOI:** 10.7759/cureus.4023

**Published:** 2019-02-06

**Authors:** Nikhila Kethireddy, Shashank Sama

**Affiliations:** 1 Internal Medicine, University of Connecticut, Farmington, USA

**Keywords:** cerebral sinus venous thrombosis (csvt), acute mastoiditis, anticoagulation, dural venous sinuses

## Abstract

Cerebral sinus venous thrombosis (CSVT) is a rare complication of acute mastoiditis with a declining incidence in the post-antibiotic era. In the adult population its incidence ranges from three to four cases per million. Here we present a case of a 47-year-old female with triple negative breast cancer on chemotherapy who underwent a molar tooth extraction, which was followed two weeks later by the sudden onset of left-sided frontotemporal headache radiating down the face, left ear fullness with associated hearing loss, toothache, and left orbital pain. Imaging studies performed included magnetic resonance imaging (MRI) as well as magnetic resonance venography (MRV), both of which showed thrombosis of the left transverse sinus, sigmoid sinus as well as the internal jugular vein, which was consistent with a diagnosis of cerebral sinus venous thrombosis. Following the diagnosis, the patient was managed with anti-coagulation and antibiotics, which resulted in improvement of her symptoms. This case highlights the need to be vigilant in patients with acute mastoiditis for the above clinical syndrome in order to promptly diagnose this rare complication and avoid life-threatening consequences.

## Introduction

Cerebral sinus venous thrombosis (CSVT) is a rare complication of acute mastoiditis with declining incidence in the post-antibiotic era [1]. Clinical presentation can vary; however, one case study reports that ipsilateral headache was present in 90% of patients found to have CSVT [1]. When clinical suspicion is high, magnetic resonance imaging (MRI) brain, magnetic resonance venogram (MRV) should be pursued as initiation of anti-coagulation and antibiotics is imperative to reduce the morbidity and mortality [1-3]. We present a case of a 47-year-old female who developed unilateral left-sided headache, aural fullness with hearing loss, painful ocular movements with visual changes and was found to have acute otomastoiditis complicated by thrombosis of the left transverse sinus, sigmoid sinus, and internal jugular vein.

## Case presentation

**Figure 1:**
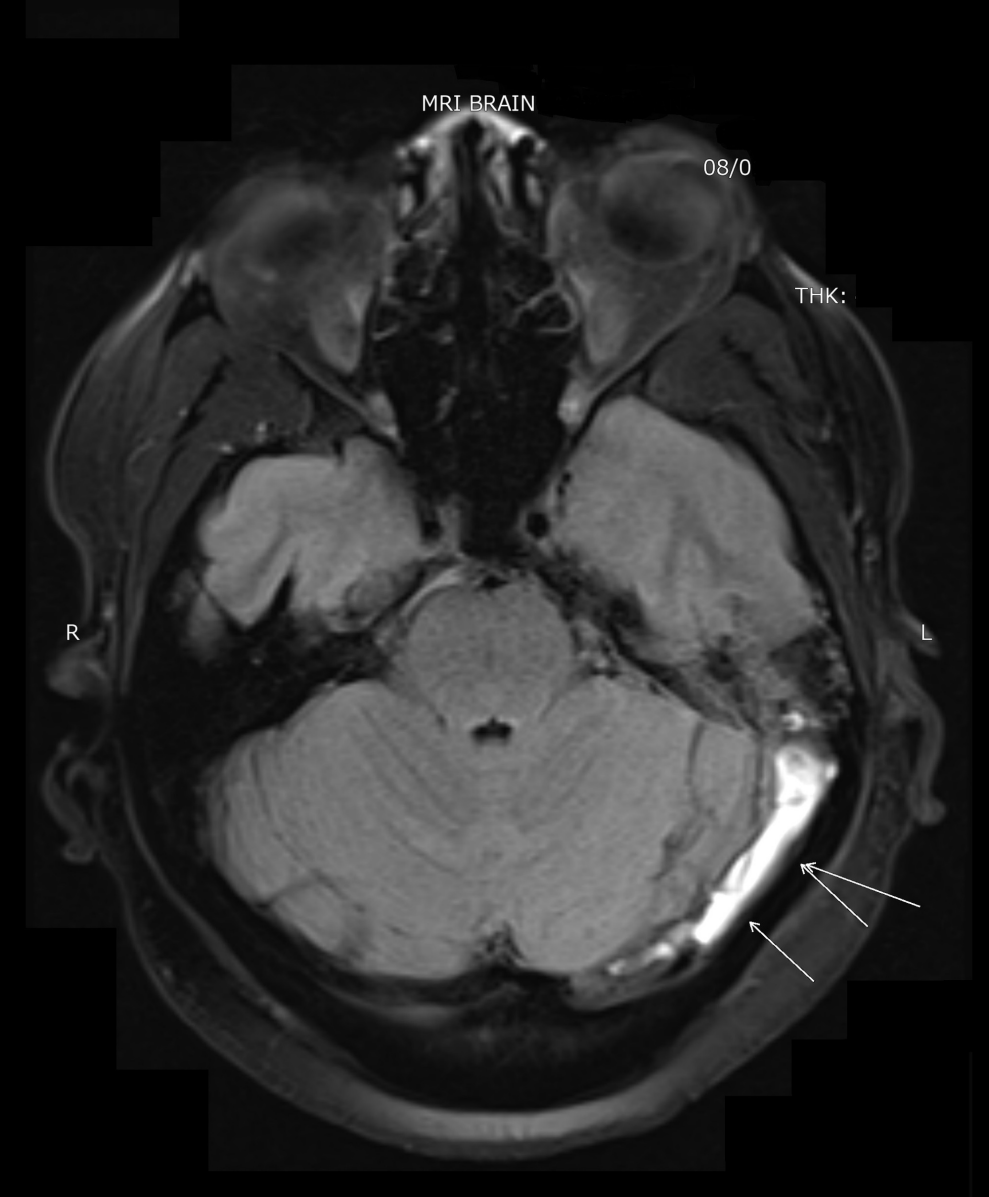
Magnetic resonance imaging of the brain showing thrombosis of the left transverse sinus.

**Figure 2:**
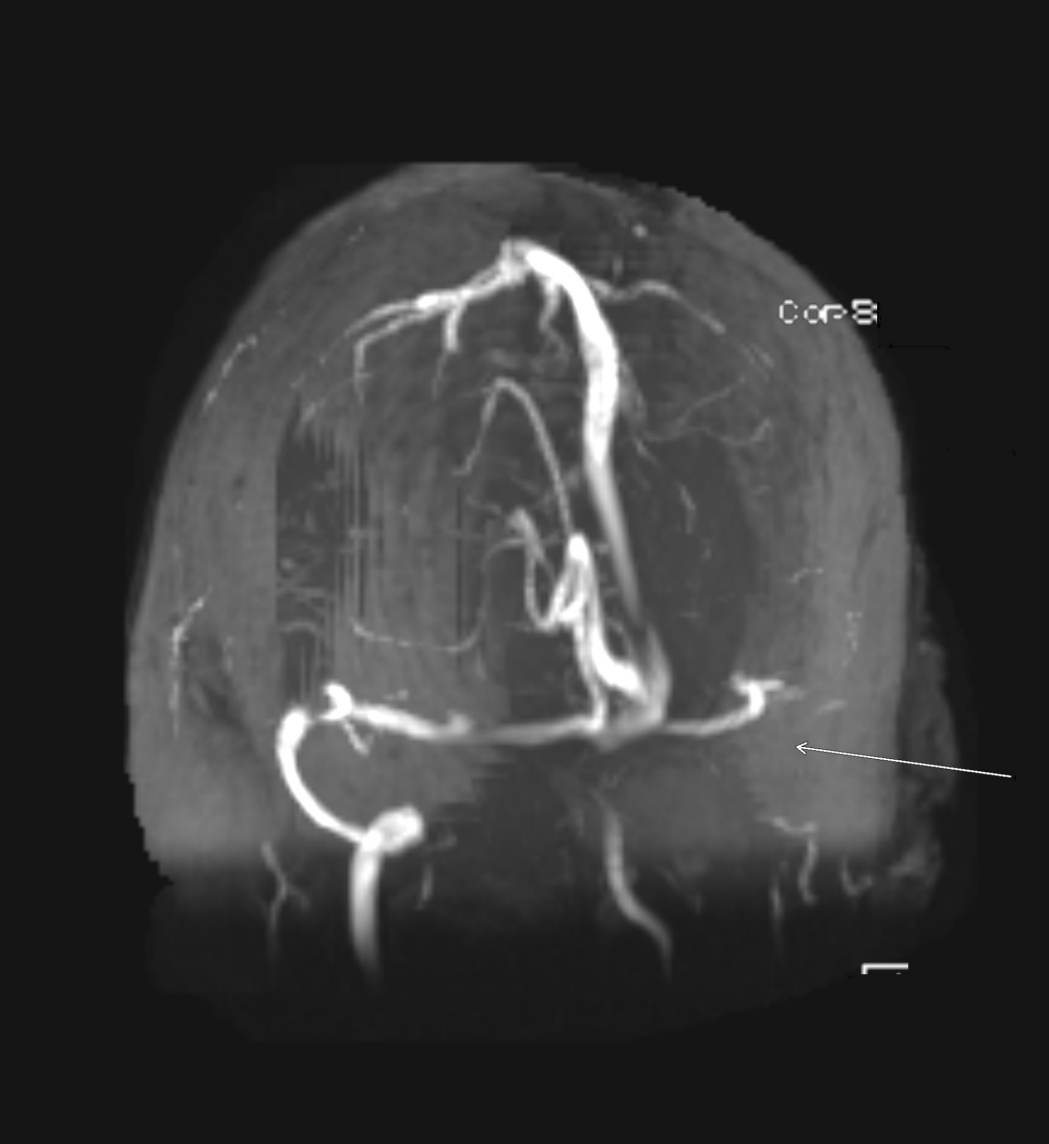
Magnetic resonance venography of the brain demonstrating occlusion of the left transverse, sigmoid sinus, and internal jugular vein (arrow).

**Figure 3:**
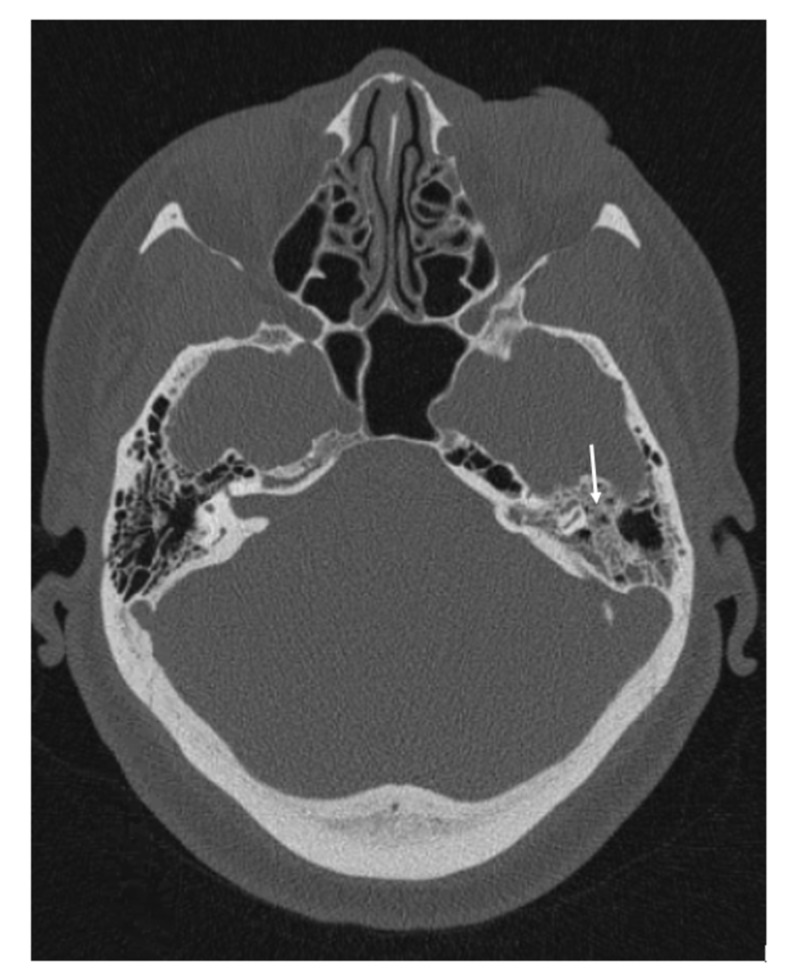
Computed tomography scan of the internal auditory canal (IAC) without intravenous (IV) contrast demonstrating fluid in the middle ear and multiple left mastoid sinus air cells (white arrow) consistent with otomastoiditis.

A 47-year-old female with triple negative right-sided breast cancer on carboplatin and paclitaxel chemotherapy, underwent a molar tooth extraction for toothache with concern for abscess. The procedure was uneventful until about two weeks after when she started to develop toothache, with sudden left-sided frontotemporal headaches radiating down her face, ear and neck, left ear fullness associated with hearing loss, and left orbital pain with blurry vision. She returned to her dentist and was given 10 days of amoxicillin for suspected sinus infection. Her symptoms worsened and she presented to emergency department (ED). In the ED she was afebrile and hemodynamically stable. Her physical exam was remarkable for left conjunctival injection with tenderness along the left side of the orbit and pain with lateral/upward/downward gaze ocular movements. Her right eye was normal in appearance. She had reduced hearing in her left ear when compared to her right, mild mastoid tenderness on the left with no edema or fluctuance with serous amber colored effusion in the left middle ear with no evidence of otitis media. The tympanic membranes bilaterally appeared intact with no effusion in the right middle ear. Her neurological exam was unremarkable with no focal neurological deficits. The rest of her physical exam was unremarkable. Initial labwork was significant for an elevated white count of 11.4 cells/mm3 (4-10.5), and computed tomography (CT) of the head did not show any abnormalities. Given her aural fullness and hearing loss, magnetic resonance imaging (MRI) of the brain and internal auditory canal was performed, which showed thrombosis of the left transverse and sigmoid sinus (Figure 1). A magnetic resonance venogram (MRV) was performed as per neurology recommendations, which re-demonstrated occlusion of the left transverse sinus, sigmoid sinus, and internal jugular vein (Figure 2). A CT scan of the internal auditory canal performed at the time showed fluid in the middle ear and multiple left mastoid sinus air cells, consistent with acute otomastoiditis (Figure 3). As the imaging helped to clarify the diagnosis, unfractionated heparin drip and broad spectrum antibiotics with vancomycin and cefepime were immediately initiated and later changed to meropenem and cefepime for the acute mastoiditis to cover Gram positive and Gram negative organisms. She was evaluated by an ear, nose and throat (ENT) specialist and no surgical intervention was indicated. Her symptoms dramatically improved and she was transitioned to subcutaneous enoxaparin, continued on intravenous (IV) antibiotics mentioned above and seen in follow-up with complete resolution of symptoms.

## Discussion

Cerebral sinus venous thrombosis (CSVT) is a rare form of venous thromboembolism (VTE) in the adult population, with an incidence of three to four cases per million and greater predominance seen in women with a 3:1 ratio when compared to men [2,4]. Middle ear infections such as acute otitis media and acute mastoiditis continue to be a huge risk factor for CSVT with mortality that was considered to be 100% in the pre-antibiotic era [2]. It is more frequently seen in young adults in developing countries due to the lack of antibiotics and resources [2].

Infections involving the middle ear structures such as acute otitis and mastoiditis are infrequently complicated by thrombosis of the sigmoid and transverse sinuses, which are contiguous structures [3, 4]. Acute otitis and mastoiditis are major risk factors for CSVT due to the propagation of infection from the small venules draining the mastoid cavity into the sigmoid sinus causing direct spread of the inflammatory process [5]. This further leads to the occlusion of cerebral veins and dural venous sinuses causing a delay in cerebrospinal fluid (CSF) absorption, which in turn causes elevated venous pressure thereby increasing intracranial pressure also known as intracranial hypertension [2, 4]. The occlusion of the veins also leads to swelling of the brain and venous infarction [4].

Other risk factors to keep in mind include oral contraceptives, puerperium, and head injury or direct injury during neurosurgical procedures [4]. Women on oral contraceptives and patients with active cancer are in a pro-thrombotic state, which further elevates the risk of cerebral sinus thrombosis [4]. Other differentials such as brain tumors, whether primary or metastatic tumors, need to be excluded especially in patients with active cancer on therapy such as in our case.

Clinical manifestations of CSVT can vary, wherein 30% can present acutely within 48 hours of blockage, 50% present in a subacute fashion within 48 hours to 30 days, and 20% may present anytime from 30 days to six months [2]. Ipsilateral headache was present in almost 90% of adult patients found to have CSVT [2, 4]. A rare case reported by Georgiadis et al. describes a middle-aged male found to have cerebral sinus thrombosis whose only presentation was a cluster-like headache [6]. Apart from headache, those with CSVT can present with edema and tenderness over the mastoid process known as Griesinger sign, nausea, vomiting, altered mental status, seizures, focal motor deficit, diplopia, and otalgia [2,4, 5]. There have been reports in which 13.2% of patients have visual deficits likely from papilledema due to increased intracranial pressure [2,3, 5]. Ophthalmoplegia can also occur due to paralysis of oculomotor, abducens or trochlear nerves with associated eye tenderness [2, 4, 5]. If untreated, the increased intracranial hypertension can lead to life-threatening complications such as permanent blindness, status epilepticus, coma, and death from cerebral herniation [4].

When clinical suspicion is high, definitive diagnosis is essential by neuroimaging. MRI of the brain combined with MRV is the most sensitive and best modality for confirmation [4, 7]. They show low or absent flow in the venous sinuses, clot formation, which appears as an increased signal intensity in T1 and T2 images and the presence of inflammation in the brain and meninges [5, 7]. MRI can be normal in up to 30% of patients [2]. Computed tomography venography (CTV) and MR venography (MRV) have a 95% sensitivity [2]. In rare cases wherein the diagnosis is still uncertain despite MRI or CTV, CT angiography and MRI angiography may be warranted as they are 100% sensitive and specific for identifying CSVT [2, 4, 7]. Angiography clearly details the partial or complete lack of venous/sinus filling with surrounding dilated and tortuous veins also referred to as “corkscrew veins” [4, 7].

Once the diagnosis of CSVT is made, initiation of anticoagulation (AC) with heparin is imperative [2, 4]. Heparin helps to recanalize the occluded cerebral veins/sinuses, reverse the thrombotic process as well as prevent further thrombus propagation and prevent pulmonary embolism [4, 7]. In a meta-analysis, it was shown heparin initiation was associated with an absolute reduction of mortality of 13% [2].

A retrospective analysis by Brucker et al., who looked at 42 cases of CSVT with various etiologies, states that heparin is recommended and has proved to be low risk even in patients with intracranial bleeding [8]. Unfractionated heparin (UFH) or low molecular weight heparin (LMWH) are most commonly utilized; however, due to practical advantages, LMWH is recommended over UFH [2]. There is insufficient evidence regarding the use of the newer anticoagulants [7]. In individuals with transient risk factors such as infection, trauma or pregnancy, the duration of AC can be three months or three to six months [2, 7]. In those with predisposing pro-thrombotic states such as active cancer, the duration is longer, about six to 12 months [2, 7]. Endovascular thrombolysis for rapid recanalization and decompressive craniotomy can be utilized in severe life-threatening cases who do not respond to medical anticoagulant therapy [2].

Historically CSVT was thought to have a high mortality rate due to life-threatening complications; however, with the new technological advances in neuroimaging and early treatment the mortality rates have become less than 3% [2]. Prognosis in CSVT is quite favorable. Preter et al. retrospectively looked at long term outcomes in 77 patients who have been diagnosed with CSVT. He reports that 85% of patients who suffered from CSVT had no long term neurological sequelae during follow-up after 77.8 months. He also found that 14.5% who did have neurological impairment suffered from seizures, cognitive, and focal neurological deficits [9]. In those patients, 11.7% had a second CSVT attack and one unfortunately had a dural arteriovenous fistula [9]. Therefore, high clinical suspicion is required for prompt diagnosis as well as early initiation of therapy with systemic anticoagulation and antibiotics leading to reduction in morbidity and mortality [3].

## Conclusions

In summary, patients at high risk of thrombosis with risk factors mentioned above should undergo imaging with MRI/MRV/CTV to identify cerebral sinus venous thrombosis and a thorough workup is warranted to identify etiology causing CSVT. Early identification and initiation of therapy can help reduce complications and even death.
